# Development of potent antibody drug conjugates against ICAM1^+^ cancer cells in preclinical models of cholangiocarcinoma

**DOI:** 10.1038/s41698-023-00447-z

**Published:** 2023-09-16

**Authors:** Bing Zhu, Xinyan Wang, Takaya Shimura, Andrew C Huang, Nana Kong, Yujie Dai, Jianmin Fang, Peng Guo, Jie-Er Ying

**Affiliations:** 1 Cancer Hospital of the University of Chinese Academy of Sciences (Zhejiang Cancer Hospital), Hangzhou, Zhejiang 310022 China; 2 Institute of Basic Medicine and Cancer (IBMC), Chinese Academy of Sciences, Hangzhou, Zhejiang 310018 China; 3 Institute of Molecular Medicine, Hangzhou Institute for Advanced Study (UCAS), Hangzhou, Zhejiang 310000 China; 4 Department of Gastroenterology and Metabolism, Nagoya City University Graduate School of Medical Sciences, Nagoya, 467-8601 Japan; 5 MabPlex International, Yantai, Shandong 264006 China; 6 School of Life Science and Technology, Tongji University, Shanghai, 200092 China; 7 Vascular Biology Program, Boston Children’s Hospital, Boston, MA 02115 USA

**Keywords:** Targeted therapies, Bile duct cancer

## Abstract

As a highly lethal adenocarcinoma of the hepatobiliary system, outcomes for cholangiocarcinoma (CCA) patients remain prominently poor with a 5-year survival of <10% due to the lack of effective treatment modalities. Targeted therapeutics for CCA are limited and surgical resection of CCA frequently suffers from a high recurrence rate. Here we report two effective targeted therapeutics in this preclinical study for CCA. We first performed a quantitative and unbiased screening of cancer-related antigens using comparative flow cytometry in a panel of human CCA cell lines, and identified intercellular adhesion molecule-1 (ICAM1) as a therapeutic target for CCA. After determining that ICAM1 has the ability to efficiently mediate antibody internalization, we constructed two ICAM1 antibody-drug conjugates (ADCs) by conjugating ICAM1 antibodies to different cytotoxic payloads through cleavable chemical linkers. The efficacies of two ICAM1 ADCs have been evaluated in comparison with the first-line chemodrug Gemcitabine in vitro and in vivo, and ICAM1 antibodies coupled with warhead DX-8951 derivative (DXd) or monomethyl auristatin E (MMAE) elicit a potent and consistent tumor attenuation. In summary, this study paves the road for developing a promising targeted therapeutic candidate for clinical treatment of CCA.

## Introduction

Cholangiocarcinoma (CCA) is a highly lethal malignancy that occurs at various locations in the biliary tree^1^. It is the second most common primary liver malignancy after hepatocellular carcinoma, accounting for 15% of primary liver cancers, and the overall incidence is on the rise globally^2,3^. Cancers originating in the bile duct proximal to second-order ducts are classified as intrahepatic cholangiocarcinoma (iCCA), those originating between the second-order bile ducts and the insertion of the cystic duct are perihilar cholangiocarcinoma, and those originating in the epithelium distal to the insertion of the cystic duct are distal cholangiocarcinoma^4^. Perihilar cholangiocarcinoma and distal cholangiocarcinoma can be collectively referred to as extrahepatic cholangiocarcinoma (eCCA). Highly aggressive disease nature with no obvious clinical symptoms in the early stage leads to most CCA patients with the disease progressed to the advanced stage at the time of diagnosis. Meanwhile, with the lack of effective therapeutic drugs, the prognosis of CCA seriously deteriorates with 5-year overall survival rate of <10%^5^.

At present, surgical resection is the only possible curative treatment option for CCA patients. However, only a small proportion achieves the conditions for radical surgical resection, with the recurrence rate as high as 66%^6^. Besides operation, currently, there are three targeted therapeutics have been approved by the FDA for the treatment of CCA. Pemigatinib and infigratinib are two small molecule inhibitors targeting fibroblast growth factor receptor (FGFR) 2 fusion or rearrangement mutations, however, only <10% of CCA patients harboring such FGFR2 genetic alterations benefit from them^7^. Ivosidenib, another small molecule inhibitor targeting isocitrate dehydrogenase-1 (IDH1) mutation, works for <13% CCA patients carrying a IDH mutation^8^. More importantly, FGFR2 translocation or IDH1 mutation predominantly occurs in iCCA, not eCCA patients^4^. Therefore, discovering new molecular targets and developing associated targeted drugs remain a significant and unmet medical need in CCA therapy.

Antibody-drug conjugates (ADCs) are emerging tumor-targeted therapeutics with promising efficacy in treating many aggressive solid tumors including gastric and breast cancers. An ADC has a monoclonal antibody coupled with cytotoxic warheads through chemical linkers, which is able to deliver the warhead to antigen-overexpressing tumor cells, resulting in a selective tumor-killing with significantly less side effects on normal tissues and organs^9^. ADCs combine the high tumor-specificity of an antibody with the potent anti-tumor activity of the cytotoxic agents, providing a viable approach to limit the exposure of normal tissue to cytotoxic payloads, in turn, reducing off-target toxicity in patients^10^. To date, no ADC has been approved for clinical treatment of CCA. DS-8201, a blockbuster HER2-targeted ADC, is currently conducting a Phase II clinical trial of biliary tract cancer (BTC), which application is limited by the low HER2 amplification rate (5–20%) in BTC patients^11^. NCT05123482, another clinical trial in Phase I/IIa, is evaluating AZD8205 (ADC targeting B7H4) for the treatment of patients with CCA^12^. In general, such clinical trials suggest that ADC as a promising treatment modality for CCA has begun to receive attention.

In this study, we identified the cell membrane protein intercellular adhesion molecule-1 (ICAM1) as a potential molecular therapeutic target for CCA by screening a panel of cancer-associated surface antigens in combination with clinical data. ICAM1 is a transmembrane glycoprotein of the immunoglobulin superfamily. As an adhesion molecule and signal receptor, ICAM1 is involved in inflammation and wound healing, and also regulates the survival and spread of tumor cells^13^. Abnormal overexpression of ICAM1 occurs in multiple types of cancers, such as non-small cell lung cancer^14^, triple-negative breast cancer^15^, melanoma^16^, oral squamous cell carcinoma^17^, and pancreatic cancer^18^. Meanwhile, serum ICAM1 was previously identified as a prognostic biomarker for early CCA detection^19^, but its therapeutic potential has yet been explored. Based on this discovered CCA therapeutic target, two ICAM1 ADCs were designed and constructed with different chemical linkers and payloads, and then their anti-tumor efficacies were evaluated on CCA by in vitro and in vivo experiments. We further explored the biological activities of ICAM1 ADCs on the tumor microenvironment of a patient-derived xenograft (PDX) of CCA via transcriptomic profiling, providing biomechanistic insights of ICAM1 ADC treatment. Taken together, these results demonstrate that ICAM1 ADCs could be a potential treatment modality for ICAM1-expressing CCA tumors.

## Results

### Identification of ICAM1 as a CCA surface target

**Fig. 1:**
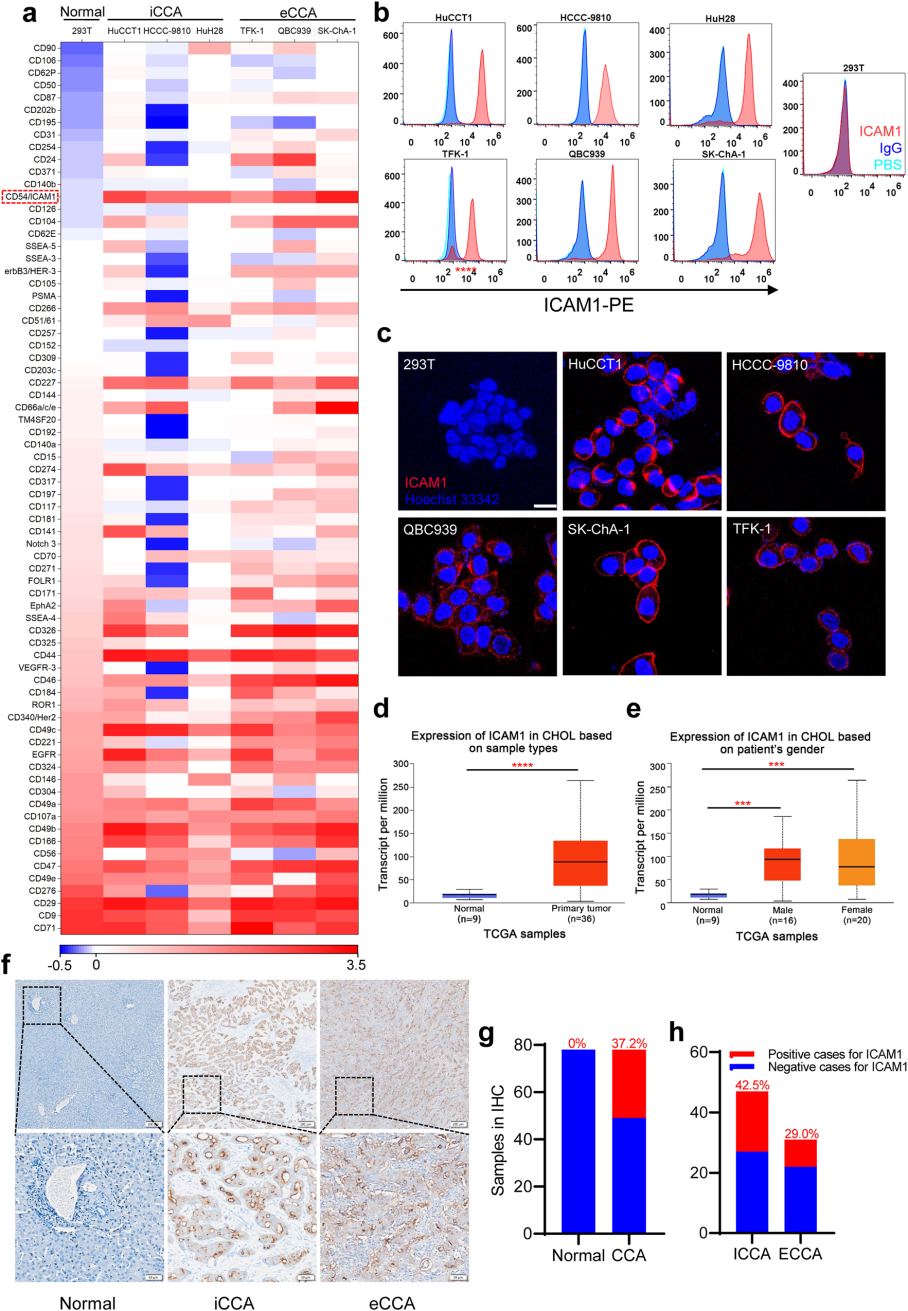
Differential overexpression of ICAM1 in human CCA. **a** Heatmap of membrane proteins expression in six human CCA cell lines and normal 293T cells. **b** Flow cytometry analysis of cell membrane expression levels of ICAM1 in six CCA cell lines and normal 293T cells. **c** IF staining of ICAM1 in human CCA and normal 293T cells. Scale bar, 20 µm. **d**, **e** ICAM1 mRNA expression levels of human CCA tumor tissues and normal bile duct tissues in TCGA samples acquired from UALCAN website. Error bars, SD. Unpaired t-test, ***P < 0.001, ****P < 0.0001. Median, quartiles, minimum, and maximum values are represented by the central line, limits of box, and ends of lines of boxplots shown. **f** Representative images of IHC staining of ICAM1 in human CCA tumor tissues (iCCA and eCCA) and normal bile duct tissues. Scale bar, 200 µm. **g** ICAM1 positive proportion in tumor and adjacent normal tissues in CCA patients (*N* = 78). **h** ICAM1 positive proportion in tumor tissues of iCCA (*N* = 47) and eCCA (*N* = 31) patients.

The first key challenge to the development of CCA-targeted ADCs is to find a suitable target antigen capable of distinguishing CCA from normal tissues, thus we performed an unbiased and quantitative screening of a panel of 72 cancer-related cell surface antigens by flow cytometry in six established human CCA cell lines^18,20^ (Fig. 1a), including three intrahepatic CCA (HuCCT1, HCCC-, and HuH28) and three extrahepatic CCA (QBC939, TFK-1, and SK-ChA-1). Then 15 candidates were found significantly overexpressed on the surface of all six CCA cells. ICAM1 was not only significantly overexpressed on the surface of six CCA cells, but also was expressed at minimum level on the surface of non-neoplastic cells (293T), comparing with other 14 candidates (Fig. 1a, b). Furthermore, we performed immunofluorescent (IF) staining of ICAM1 on CCA cells, confirming that the overexpression of ICAM1 was localized on the plasma membranes of each CCA cell lines (HuCCT1, HCCC-9810, QBC939, SK-ChA-1, and TFK-1) but was absent on normal 293T cells (Fig. 1c), making it accessible for ICAM1 ADCs.

To correlate our findings with CCA clinical data, we compared ICAM1 mRNA expression levels with human CCA tumors and normal bile duct tissues by querying the UALCAN: The University of ALabama at Birmingham CANcer data analysis Portal (http://ualcan.path.uab.edu/)^21,22^, using the database of The Cancer Genome Atlas Program (TCGA). As observed in Fig. 1d, the expression of ICAM1 was significantly upregulated in human CCA tumors (*n* = 36) compared with normal bile duct tissues (*n* = 9), in consistence with our findings in vitro. In addition, the overexpression of ICAM1 was not significantly different between male (*n* = 16) and female patients (*n* = 20) (Fig. 1e).

We further investigated ICAM1 protein expression in human CCA tumor tissues by performing immunohistochemical (IHC) staining in 78 human CCA tumor tissues (including 47 patients tumor tissues with iCCA and 37 patients with eCCA) and corresponding para-cancerous tissues (normal bile duct tissues or liver tissues). As demonstrated in Fig. 1f, ICAM1 protein was significantly overexpressed in tumor tissues of both iCCA and eCCA, but was absent in the normal human bile duct tissues. The statistical analysis showed that CCA patients with ICAM1^+^ expression in cancer tissues was as high as 37.2%, while there was no expression of ICAM1 in the normal bile duct tissues and liver tissues in the corresponding para-cancerous tissues (Fig. 1g). Furthermore, there were 42.5% of patients with iCCA and 29.0% with eCCA carrying ICAM1^+^ cancer cells, among 78 CCA patients (Fig. 1h). These findings strongly support that ICAM1 is a promising molecular target for developing CCA-targeted therapeutics.

### ICAM1 efficiently mediates antibody endocytosis in CCA cells

**Fig. 2:**
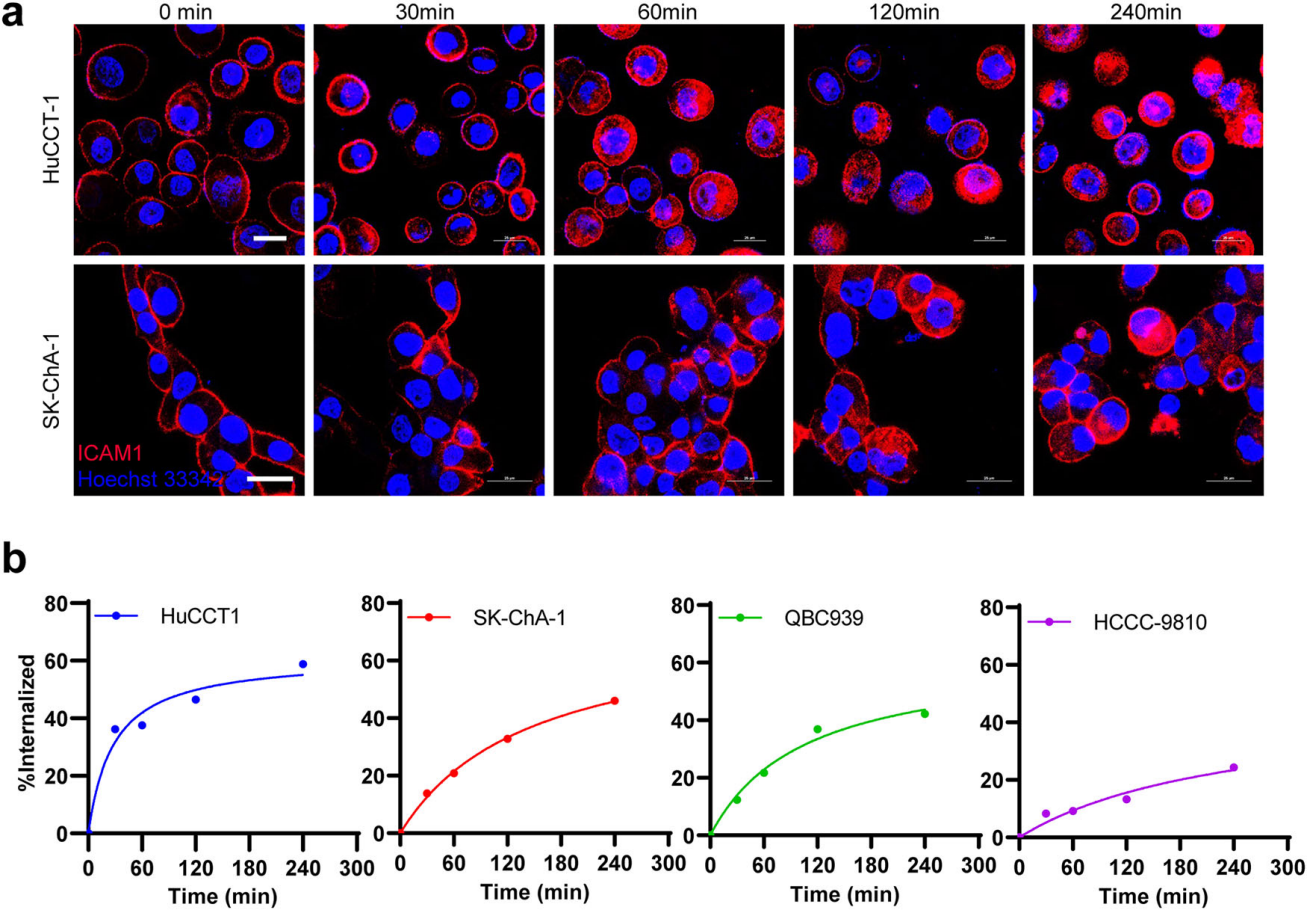
The internalization of ICAM1 antibodies mediated by membrane protein ICAM1 on the surface of human CCA cells. **a** Representative IF images showing cellular internalization of ICAM1 antibodies in human CCA cells (HuCCT1 and SK-ChA-1). Scale bar, 25 µm. **b** The internalization efficiency of ICAM1 antibodies in four human CCA cell lines (HuCCT1, SK-ChA-1, QBC939, and HCCC-9810) quantified by flow cytometry.

The target of ADCs needs to exert efficient internalization after binding to the antibody, so that the antigen-ADC complex can be transported into cytoplasm through antigen-mediated endocytosis, and then play the role of cell destruction^23^. On the contrary, an inefficient antibody endocytosis will make payloads of ADCs release and circulate outside the tumor, which not only reduces the drug efficacy, but also increases off-target toxicities^24^. To determine whether the membrane protein ICAM1 in CCA could effectively internalize its antibody into the cytoplasm through antigen-mediated endocytosis, we first observed the endocytosis phenomenon of ICAM1 protein on the surface of two CCA cell lines by immunofluorescent confocal microscopy using phycoerythrin (PE) labeled ICAM1 antibodies (Fig. 2a). Imaging results showed that the fluorescent-labeled ICAM1 antibodies were initially bound to ICAM1 antigens on the surface of HuCCT1 (iCCA) and SK-ChA-1 (eCCA) cells. Over time, ICAM1 antibodies were swiftly internalized by both CCA cells. The internalization efficiency of ICAM1 antibodies in CCA cells was quantified using an established flow cytometry assay^25^ (Fig. 2b). Calculated results showed that at the time of 240 min, the internalization efficiency of ICAM1 antibodies in HuCCT1 cells was close to 60%, and the endocytosed antibodies were more than 40% in SK-ChA-1 and QBC939 cells, while HCCC-9810 cells had a lower endocytosis rate of 24% relatively. These results showed that ICAM1 protein on the surface of CCA cells can effectively mediate antibody endocytosis, indicating that ICAM1 can be a potential ADC target for CCA.

### Design and preparation of ICAM1 ADCs

**Fig. 3:**
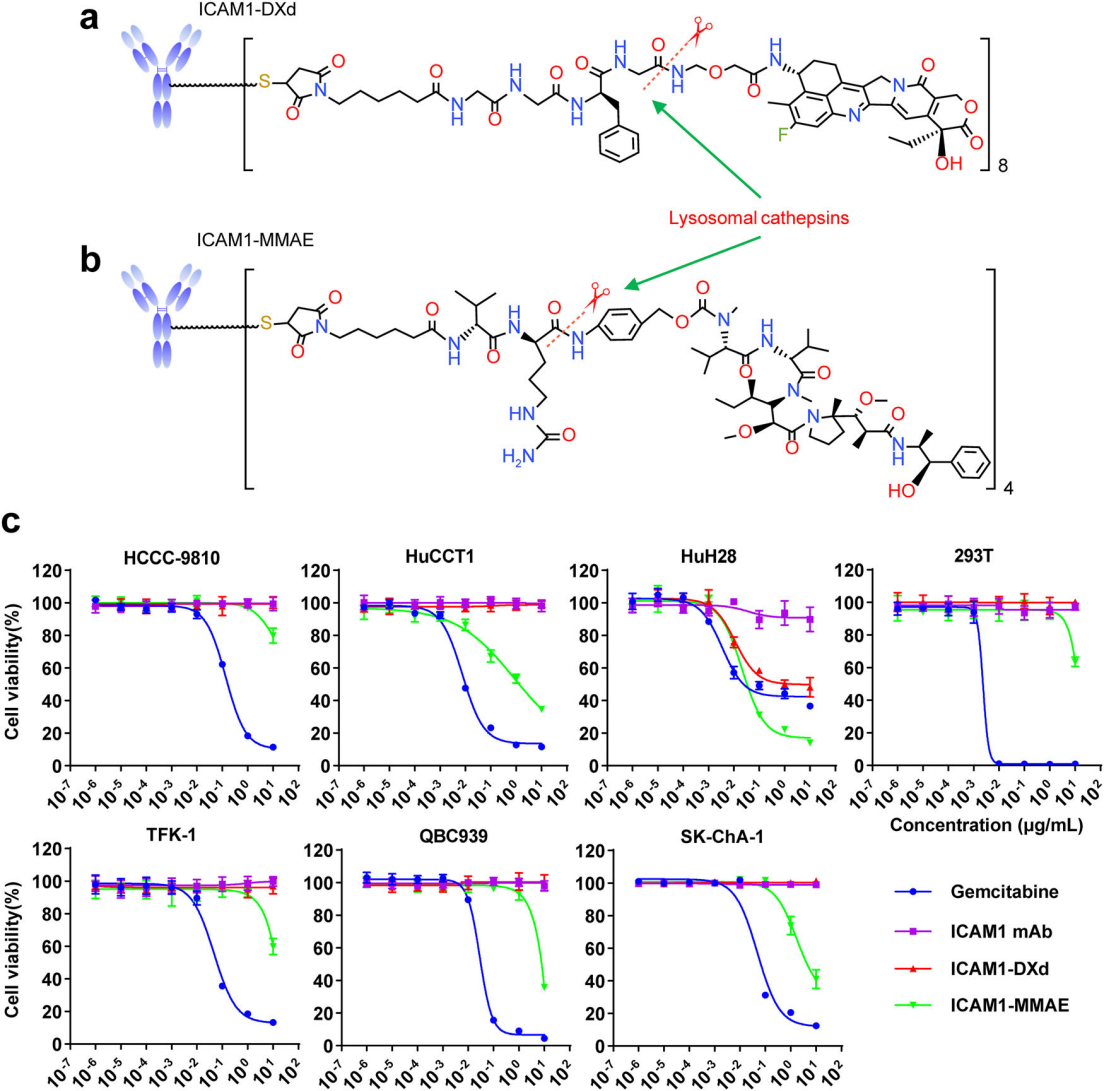
ICAM1 ADCs selectively ablating human CCA cells in vitro. **a** Schematic diagram of the structure of an ICAM1-DXd. **b** Schematic diagram of the structure of an ICAM1-MMAE. **c** In vitro cytotoxicity of ICAM1 antibody (ICAM1 mAb), two ICAM1 ADCs (ICAM1-MMAE and ICAM1-DXd), and one chemotherapy drug Gemcitabine against a panel of six human CCA cell lines and normal 293T cells.

To construct ICAM1 ADCs, we first selected a monoclonal human/mouse chimeric ICAM1 antibody^26^ containing a human constant fragment (Fc) that effectively reduces immunogenicity meanwhile provides a good human safety profile^27^. In order to optimize ADC formulation, we designed and constructed two ICAM1 ADCs with different clinically-approved linkers and warheads, one was ICAM1-DXd, which ICAM1 antibodies were conjugated to ~8 molecules of a topoisomerase I inhibitor, DXd, a DX8951 derivative, via a peptidyl spacer (Gly-Gly-Phe-Gly, GGFG) (Fig. 3a). The other was ICAM1-MMAE, which ICAM1 antibodies were coupled to ~4 molecules of monomethyl auristatin E (MMAE) with a valine-citrulline linker (Fig. 3b). The drug-to-antibody ratio (DAR) values of ICAM1-DXd and ICAM1-MMAE were characterized by hydrophobic interaction chromatography (HIC) in Supplementary Figure 1. Both linkers can be selectively cleaved by cathepsin B proteases within CCA cell endosomes/lysosomes, providing clinically-validated therapeutic benefits in treating tumor microenvironments via bystander killing effects^28^.

The reason we selected MC-VC-PAB-MMAE and MC-GGFG-DXd as our linker and payload combinations for ICAM1 ADC construction is due to the fact that they represent two different payload mechanism of actions (MOAs): MMAE works as a microtubule inhibitor suppressing cancer cell mitosis while DXd is a DNA topoisomerase I inhibitor and induces cancer cell death by generating DNA damages. Both of them has demonstrated effective efficacies against gastrointestinal cancers, which has been clinically used in two HER2 ADCs, namely RC48 and DS8201. We assessed the binding abilities of ICAM1-DXd and ICAM1-MMAE in comparison with their parent ICAM1 antibody by using flow cytometry. As shown in Supplementary Figure 2, both ICAM1 ADCs demonstrated the same binding ability with their parent ICAM1 antibody in two human CCA cell lines (SK-ChA-1 and TFK-1), suggesting that conjugating ADC linkers and payloads on ICAM1 antibody via its inter-chain disulfide cysteine does not obviously affect its binding ability. Moreover, we also evaluated the endocytosis efficiency of the ICAM1 monoclonal antibody (clone: R6.5) used to construct ADCs in four human CCA cell lines (QBC939, SK-CHa-1, TFK-1, and HCCC-9810). As shown in Supplementary Figure 3, ~20–40% of ICAM1 monoclonal antibodies were readily internalized by four human CCA cell lines after 240 min incubation.

We evaluated the cytotoxicity of ICAM1 ADCs in vitro by quantifying the half maximum inhibitory concentrations (IC50s) of each ADC in ablating human CCA cells and normal 293T cells. The first-line chemodrug Gemcitabine and unconjugated ICAM1 monoclonal antibodies were used as positive controls. From the results of quantified IC50s of ICAM1 ADCs and Gemcitabine, we found that ICAM1-DXd did not significantly inhibit CCA cells and normal 293T cells in vitro at the set drug concentrations (0–10 µg/mL), while ICAM1-MMAE did potently ablate CCA cells at the highest concentration of 10 µg/mL. Such in vitro cytotoxicity differences between ICAM1-DXd and ICAM1-MMAE were predictable since DXd is well-known for the remarkably lower cytotoxicity than MMAE based on their different mechanisms of actions^29^. Neither ICAM1-DXd or ICAM1-MMAE showed any toxicities against normal 293T cells. In comparison, Gemcitabine markedly killed non-neoplastic 293T cells at the dosage of 100 times lower than ICAM1-MMAE (Fig. 3c). Considering the fact that the molecular mass of ICAM1 ADCs (~150,000 Dalton) is more than 500 times larger than Gemcitabine (263 Dalton), these results suggest that ICAM1 ADCs can work more effective and selective than Gemcitabine, warranting further investigation in animal studies (Table 1).

**Table 1:** IC50s (µM) for different drugs against different cell lines in vitro.

Cell line	Drug			
	Gemcitabine	ICAM mAb	ICAM1-DXd	ICAM1-MMAE
SK-ChA-1	190.46	–	–	28.95
QBC939	134.89	–	–	43.81
TFK-1	220.67	–	–	78.53
HuH28	100.82	–	8.43	0.20
HuCCT1	37.83	–	–	8.52
HCCC-9810	621.59	–	–	329.36
293T	8.78	–	–	87.23

We further evaluated the bystander killing effects of ICAM1-DXd and ICAM1-MMAE utilizing an established co-culture system of TFK-1 (ICAM1^+^)/RBE (ICAM1^-^) cells^30^. Non-specific IgG-MMAE and IgG-DXd were used as controls. As shown in Supplementary Figure 4, both ICAM1-DXd and ICAM1-MMAE potently ablated TFK-1 (ICAM1^+^) cells via antigen-specific targeting. Moreover, ICAM1-DXd also ablated 78% of RBE (ICAM1^-^) cells, which was significantly higher than that of ICAM1-MMAE (33%), indicating that the GGFG quadrapeptide linker of ICAM1-DXd can mediate a more potent bystander killing effect than the VC dipeptide linker of ICAM1-MMAE in vitro.

### ICAM1 ADCs selectively target CCA tumors in vivo

**Fig. 4:**
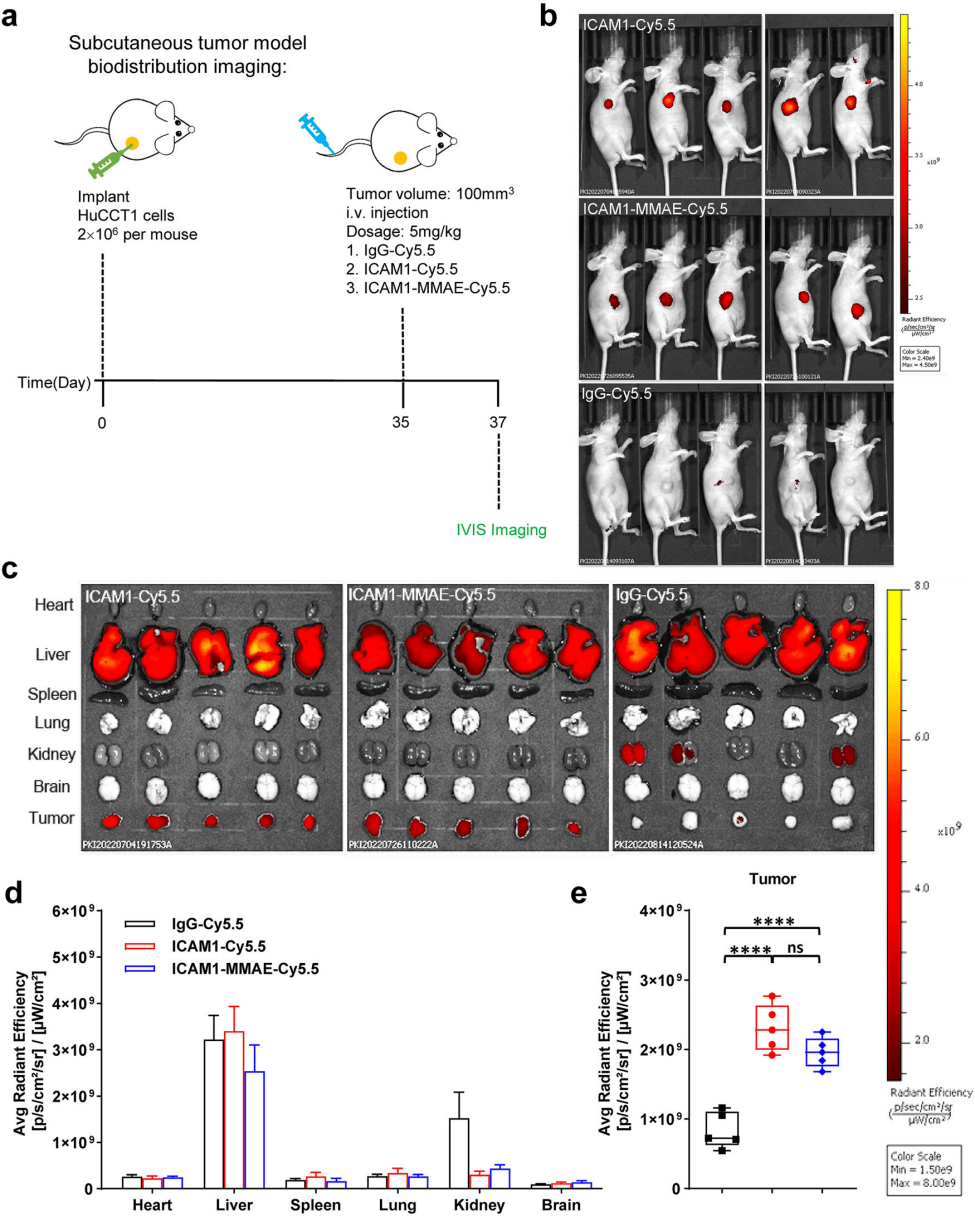
Tumor-specificity and biodistribution of ICAM1 antibody and ICAM1-MMAE in vivo. **a** Schematic design of CCA biodistribution study in a CCA tumor (HuCCT1) xenografted model. **b** In vivo NIR fluorescent images of nude mice at 48 h after the administration of IgG-Cy5.5, ICAM1-Cy5.5, and ICAM1-MMAE-Cy5.5 (*N* = 5 per group). **c** Representative ex vivo NIR fluorescent images of HuCCT1 tumor and six major organs (heart, liver, spleen, lung, kidney, and brain). **d** Quantified normal organ distribution of IgG-Cy5.5, ICAM1-Cy5.5, and ICAM1-MMAE-Cy5.5 (*N* = 5). **e** Quantified HuCCT1 tumor accumulation of IgG-Cy5.5, ICAM1-Cy5.5, and ICAM1-MMAE-Cy5.5 (*N* = 5 per group). Unpaired t-test, ****P < 0.0001, ns not significant. Error bars, SD. Median, quartiles, minimum, and maximum values are represented by the central line, limits of box, and ends of lines of boxplots shown.

We next evaluated the tumor specificity and biodistribution of ICAM1 ADCs in vivo. ICAM1 antibody and one ICAM1 ADC (ICAM1-MMAE) were labeled with a near-infrared fluorescent dye Cy5.5, respectively, and one untargeted mouse IgG was used as a control. A CCA tumor (HuCCT1) xenografted nude mouse model was used to determine the distribution of fluorescently labeled antibodies and ICAM1 ADC via intravenous route (Fig. 4a). In vivo near-infrared (NIR) fluorescent imaging was performed on treated mice using an IVIS Lumina III at 48 h post-injection. In vivo imaging results showed that, in comparison with non-targeted IgG-Cy5.5, ICAM1-Cy5.5 and ICAM1-MMAE-Cy5.5 had significantly higher accumulation at CCA tumor sites (Fig. 4b). Statistical analysis confirmed that fluorescent signals of ICAM1-Cy5.5 and ICAM1-MMAE-Cy5.5 accumulated at the tumor site were more than 2-fold higher than that of IgG-Cy5.5 (Fig. 4e). We further detected the distribution of ICAM1-Cy5.5, ICAM1-MMAE-Cy5.5, and IgG-Cy5.5 in the six major organs of heart, liver, spleen, lung, kidney, and brain. As shown in Fig. 4c, d, liver was the major non-tumor accumulation site of ICAM1-Cy5.5 and ICAM1-MMAE-Cy5.5 as well as non-targeted IgG-Cy5.5. Non-specific liver uptake of other antibodies and ADCs has been reported both in humans and in experimental animals^31^, which is probably due to the fact that liver-resident Kupffer cells non-specifically recognize antibodies and ADCs through their Fc receptors (FcRs) and trigger antibody-dependent cellular phagocytosis. These results validate that ICAM1 ADCs can exhibit similar tumor targeting activity as the ICAM1 antibody, which was not affected by coupling with ADC linkers and payloads.

### ICAM1 ADCs potently ablate CCA tumors in vivo

**Fig. 5:**
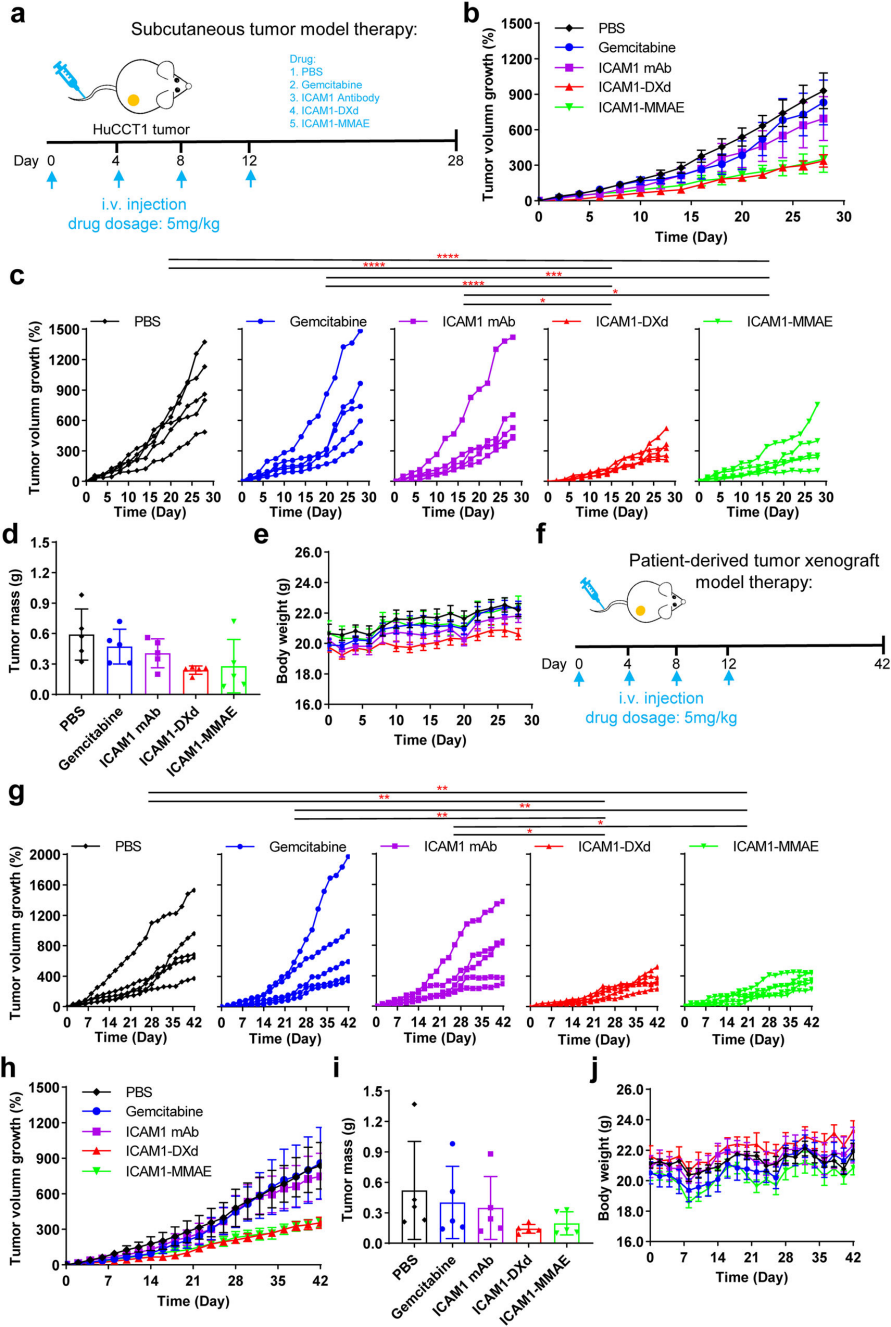
Tumor-specific efficacy of ICAM1 ADCs on CCA tumors in vivo. **a** Schematic design of in vivo efficacy for ICAM1 ADCs in a CCA tumor (HuCCT1) xenografted model. **b** Tumor progression in the CCA tumor (HuCCT1) xenografted model treated with PBS, ICAM1 antibody (ICAM1 mAb), Gemcitabine, ICAM1-DXd, or ICAM1-MMAE, respectively, monitored by tumor volume measurement (*N* = 5 per group). Error bars, SEM. **c** Statistical analysis of tumor progression difference between various treatment groups in the CCA tumor (HuCCT1) xenografted model. Two-way ANOVA, *P < 0.05, **P < 0.01, ***P < 0.001, ****P < 0.0001. **d** Tumor mass (at day 28) and **e** mouse bodyweight of mice in the HuCCT1 xenografted model. Error bars, SD. **f** Schematic design of in vivo efficacy for ICAM1 ADCs in PDX model. **g** Statistical analysis of tumor progression difference in the PDX model treated with PBS, ICAM1 mAb, Gemcitabine, ICAM1-DXd or ICAM1-MMAE, respectively (*N* = 5 per group). Two-way ANOVA, *P < 0.05, **P < 0.01. **h** Tumor progression in PDX model was monitored by tumor volume measurement (*N* = 5 per group). Error bars, SEM. **i** Tumor mass (at day 28) and **j** mouse bodyweight of mice in the PDX model. Error bars, SD.

We next evaluated the in vivo efficacy of two ICAM1 ADCs using the same CCA tumor xenograft model. ICAM1-MMAE and ICAM1-DXd were administered at 5 mg/kg once every 3 days, while control groups were treated with PBS, ICAM1 antibody (ICAM1 mAb) and Gemcitabine, respectively, at the same dosage (Fig. 5a). As seen from Fig. 5b, c, the CCA tumor growth was remarkably inhibited after ICAM1-MMAE or ICAM1-DXd treatment, in comparison with control groups throughout the course of treatment. The anti-tumor activity of two ADCs was also determined by weighing the tumor mass at the end point, and the inhibitory efficiency of tumor weight by ICAM1-DXd and ICAM1-MMAE were 59% and 53% of PBS, respectively (Fig. 5d). In comparison, Gemcitabine showed almost no inhibitory effect on CCA tumors, while ICAM1 antibodies showed a certain inhibitory effect on tumor growth, but was significantly less effective than ICAM1-MMAE and ICAM1-DXd treatments. At the end of the in vivo study, we collected mouse blood samples from each group via cardiac puncture and tested the levels of two established serum biomarkers of liver toxicity, aspartate aminotransferase (AST) and alanine aminotransferase (ALT). As shown in Supplementary Figure 5a, among two ICAM1 ADCs, neither of them induced any significant elevation in either AST or ALT levels relative to the PBS group. Similarly, we evaluated the renal toxicity of ICAM1-DXd and ICAM1-MMAE by measuring creatinine and blood urea nitrogen (BUN) levels and we observed no renal toxicity of the two ICAM1 ADCs. And we also collected mouse livers and kidneys for H&E staining. As shown in Supplementary Figure 5b, compared with PBS, the liver and kidney of mice treated with ICAM1-DXd or ICAM1-MMAE, did not appear any obvious pathological changes, suggesting both of ICAM1 ADCs are relatively safe and well tolerated in animals.

Furthermore, we evaluated the therapeutic efficacy of two ICAM1 ADCs (ICAM1-MMAE and ICAM1-DXd) in comparison with non-targeting isotype ADCs, IgG-MMAE (DAR: 4) and IgG-DXd (DAR: 8). In the same CCA tumor xenografts (Supplementary Figure 6), both ICAM1 ADCs exhibited significantly higher potency against CCA tumors than their non-targeting counterparts, suggesting that ICAM1 ADCs can mediate their anti-tumor activity in a tumor-specific manner.

To evaluate ICAM1 ADCs in a more clinically relevant setting, we further utilized a CCA patient-derived tumor xenograft (PDX) model with the same treatment regimen (Fig. 5f). This PDX model features a low ICAM1 expression (Supplementary Figure 7), which is about 500-fold lower than that of HuCCT1 tumor xenograft. As shown in Fig. 5g, h, in comparison with control groups, the CCA tumor growth rate in the ICAM1 ADC-treated groups showed a significantly decreasing trend. The anti-tumor activity of ICAM1-DXd and ICAM1-MMAE on tumor growth at the end of treatment were 73% and 62%, respectively, in comparison with the PBS control group (Fig. 5i). Moreover, ADC dosage at 5 mg/kg did not affect mouse bodyweight in both ICAM1-MMAE and ICAM1-DXd-treated groups (Fig. 5e, j). Overall, both ICAM1 ADCs showed significant tumor growth inhibition in both in vivo treatment models, and ICAM1-DXd is consistently more effective than ICAM1-MMAE in both CCA tumor models.

### ICAM1-DXd attenuates tumor growth via type I interferon signaling pathway

**Fig. 6:**
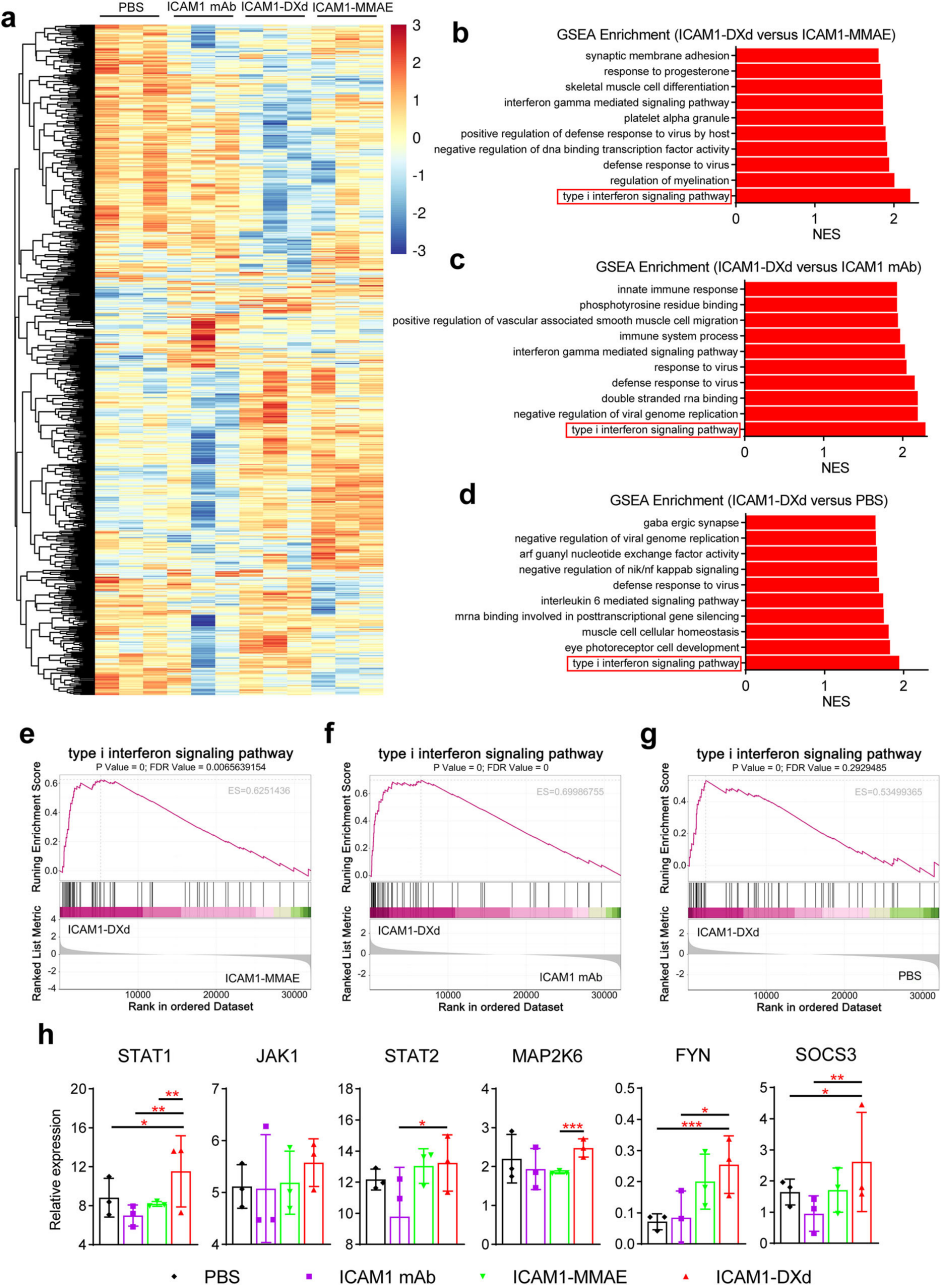
ICAM1-DXd activating type I interferon signaling in PDX model. **a** Heatmap of significant gene expression in PDX model with different treatment (PBS, ICAM1 mAb, ICAM1-DXd, ICAM1-MMAE), analyzed by RNA-seq (*N* = 3 per group). **b**–**d** Gene Set Enrichment Analysis (GSEA) of RNA-seq data from PDX model with different treatment (PBS, ICAM1 mAb, ICAM1-DXd, ICAM1-MMAE), showing the top 10 most significantly upregulated pathways in ICAM1-DXd, comparison to other treatment groups. NES, normalized enrichment score. **b** ICAM1-DXd versus ICAM1-MMAE. **c** ICAM1-DXd versus ICAM1 mAb. **d** ICAM1-DXd versus PBS. *N* = 3 per group. **e**–**g** Changes of type I interferon signaling pathway was performed in ICAM1-DXd treated PDX tumor tissues versus other treatment groups via GSEA. FDR, false discovery rate. ES, enrichment score. **e** ICAM1-DXd versus ICAM1-MMAE. **f** ICAM1-DXd versus ICAM1 mAb. **g** ICAM1-DXd versus PBS. **h** Six genes with significant differences in the expression of type I interferon signaling pathway in PDX tumor tissues of different treatment groups. Negative binomial distribution, *P < 0.05, **P < 0.01, ***P < 0.001. Error bars, SD.

We observed an interesting contradictory phenomenon that ICAM1-DXd showed a very weak cytotoxicity on CCA cell lines in vitro, whereas it mediated more potent tumor attenuation than ICAM1-MMAE in vivo, suggesting the potency of ICAM1-DXd may arise from its multifaceted biological activities instead of merely cytotoxicity. To elucidate its underlying biomechanism, we performed a transcriptomic RNA sequencing (RNA-seq) to profile the signaling pathways and key genes affected by ICAM1-DXd treatment in PDX tumor tissues (Fig. 6a). Notably, Gene Set Enrichment Analysis (GSEA) revealed that type I interferon signaling pathway was the most enriched one in PDX tumor tissues treated with ICAM1-DXd in comparison with PBS, ICAM1 mAb, and ICAM1-MMAE (Fig. 6b–g). We further investigated the key genes involved in ICAM1-DXd activated type I interferon signaling cascades. As shown in Fig. 6h, STAT1, JAK1, STAT2, MAP2K6, FYN, SOCS3 are six most significantly upregulated genes in type I interferon signaling pathway activated by ICAM1-DXd. STAT1 is an important upstream regulatory gene in type I interferon signaling pathway, and upregulation of type I interferon expression in tumors has been shown to facilitate immunostimulatory effects, which can further enhance anti-tumor effects after induction of anti-tumor immune responses^32^. The above RNA-seq analysis showed that ICAM1-DXd treatment not only directly ablates CCA cells but also improves tumor immune microenvironment via significantly upregulating the type I interferon signaling pathway in a PDX model.

## Discussion

To date, CCA remains a devastating malignancy without effective therapeutics in the clinic. To address this challenge, we report two rationally designed ADCs, ICAM1-DXd and ICAM1-MMAE, capable of serving as the potent targeted therapeutic candidate for CCA treatment. In comparison with existing CCA therapeutics, our ICAM1 ADCs feature following advantages: first, we identified ICAM1 as an effective cell membrane protein target for CCA, which can be broadly used for developing many targeting therapeutic modalities including ADCs, CAR-T/NK/macrophage cell therapies, and oncolytic viruses. Second, our PDX results strongly support that ADCs with protease-cleavable linkers facilitated potent bystander killing effects in CCA tumors. Third, our transcriptomic profiling results provide new biological insights that ICAM1-DXd affects CCA tumor immune microenvironment mainly through upregulating type I interferon signaling pathway.

To our knowledge, our results provide the first experimental evidence of utilizing ICAM1 as an effective ADC target for CCA. In this study, we repeatedly confirmed that tumor-selective overexpression of ICAM1 at protein and mRNA levels in well-recognized CCA cell lines and clinically-relevant tumor tissues. Importantly, the CCA-specificity of ICAM1 is higher than many common cancer targets such as ROR1, HER2, EGFR, VEGFR, and P-selectin (Fig. 1). Moreover, the efficient ICAM1 antigen-mediated endocytosis of its antibodies was directly visualized and its internalization rate was quantified in four CCA cell lines (Fig. 2), which can be conducive to assist ADC warheads to cross CCA cell membrane in order to exert cytotoxic effects^33^.

In addition to target selection, we also optimized ADC linkers and warheads for CCA treatment by utilizing an unbiased and quantitative screening. We first constructed two ICAM1 ADCs with different combinations of clinically-effective linkers and warheads and evaluated their efficacies against CCA cells in vitro and in vivo. One of the cytotoxic warheads, MMAE, as a dolastatin-10 peptide derivative, with potent antimitotic activity that inhibites cell division by blocking the tubulin polymerization, which is the most commonly-used warhead in clinically-approved ADCs and facilitates potent efficacy against other gastrointestinal cancer (gastric cancer) in RC48, a HER2-targeted ADC^34^. For the other ICAM1 ADC, we selected DXd as the second warhead due to its different drug mechanism of action. As a derivative of Irinotecan, DXd is a topoisomerase I inhibitor that mediates anti-tumor activity by inhibiting DNA transcription and replication of tumor cells, which is also clinically-used in DS8201, another HER2-targeted ADC^35^. Both ICAM1 ADCs have bystander killing effects by utilizing protease-cleavable linkers of dipeptide (Val–Cit) or quadrapeptide (Gly-Gly-Phe-Gly), which can rapidly release ADC warheads from ICAM1^+^ CCA cells to surrounding ICAM1^-^ tumor cells and stromal cells including tumor-associated macrophages or fibroblasts.

Our preclinical data strongly supports that ICAM1-DXd is a promising ADC candidate for CCA, which can be explained by two advantages, the proportion of beneficiaries and the drug efficacy. First, IHC staining revealed that ICAM1 was overexpressed in at least 37% of 79 CCA patients. Further subdividing the disease subtypes of CCA patients showed that 42.5% of iCCA patients overexpressed ICAM1, while eCCA patients with 29%. These CCA patients overexpressing ICAM1 can benefit from ICAM1 ADCs. Secondly, through two in vivo models, ICAM1-DXd and ICAM1-MMAE showed potent anti-tumor activities in comparison with the first-line chemodrug Gemcitabine. But the efficacy of ICAM1-DXd is consistently better than that of ICAM1-MMAE. Part of the reason attributes to the different properties of free warheads after cathepsin B cleavage. It has been known that free MMAE has poor membrane penetration and weak bystander killing ability, while DXd is lipid-soluble and can freely penetrate the cell membrane composed of lipids, which can also damage ICAM1^-^ cancer and stroma cells in solid tumors and exert a powerful bystander killing effect^30^. Moreover, ICAM1-DXd can further enhance the anti-tumor immunity by significantly upregulating the type I interferon signal pathway.

In summary, we identified ICAM1 could be a molecular therapeutic target for CCA and validated its therapeutic potential by constructing two rational ICAM1 ADCs, ICAM1-MMAE and ICAM1-DXd. Systematic ADC efficacy studies revealed ICAM1-DXd as an optimized CCA-targeted ADC formulation warranting further investigations in more clinically-oriented settings. Our transcriptomic RNA-seq analysis further indicated that ICAM1-DXd also activates anti-tumor immunity in PDX models. ICAM1-DXd can mediate potent and sustained CCA tumor attenuations through comprehensive and synergistic benefits from ADC treatment. Together, our study provides critical insights into the development of CCA-targeted ADC candidates.

## Methods

### Antibodies, reagents, and chemicals

Phycoerythrin (PE)-conjugated mouse anti-human antibodies against 72 cancer target candidates (CD90, Cat#328110; CD106, Cat#305806; CD62P, Cat#304906; CD50, Cat#330005; CD87, Cat#336906; CD202b, Cat#334206; CD195, Cat#321606; CD31, Cat#303106; CD254, Cat#347504; CD24, Cat#323206; CD371, Cat#353604; CD140b, Cat#323606; CD54, Cat#353106; CD126, Cat#352804; CD104, Cat#327808; CD62E, Cat#336008; SSEA-5, Cat#355204; SSEA-3, Cat#330312; HER-3, Cat#324706; CD105, Cat#323206; PSMA, Cat#342504; CD266, Cat#314004; CD51/61, Cat#304406; CD257, Cat#366506; CD152, Cat#349906; CD309, Cat#393004; CD203c, Cat#324606; CD227, Cat#355604; CD144, Cat#348506; CD66a/b/c, Cat#342304; TM4SF20, Cat#367204; CD192, Cat#357206; CD140a, Cat#323506; CD152, Cat#349906; CD274, Cat#329706; CD317, Cat#127104; CD197, Cat#353204; CD117, Cat#375206; CD181, Cat#320608; CD141, Cat#344104; Notch 3, Cat#345406; CD70, Cat#355104; CD271, Cat#345106; FOLR1, Cat#908304; CD171, Cat#371604; EphA2, Cat#356804; SSEA-4, Cat#330406; CD326, Cat#324206; CD325, Cat#350805; CD44, Cat#397504; VEGFR-3, Cat#356204; CD46, Cat#352402; CD184, Cat#306506; ROR1, Cat#357804; CD340, Cat#324406; CD49c, Cat#343803; CD221, Cat#351806; EGFR, Cat#352904; CD324, Cat#324406; CD146, Cat#361006; CD304, Cat#354504; CD49a, Cat#328304; CD107a, Cat#328608; CD49b, Cat#359308; CD166, Cat#343904; CD56, Cat#362508; CD47, Cat#323108; CD49e, Cat#328010; CD276, Cat#351004; CD29, Cat#303004; CD9, Cat#312106; CD71, Cat#334106), PE mouse IgG1 (Cat#400114), purified anti-human CD54 Antibody (Cat#322702), PE anti-mouse IgG1 Antibody (Cat#406608) and PE anti-human IgG Fc (Cat#410708) were purchased from BioLegend (San Diego, CA, USA). Gemcitabine, anti-ICAM1 antibody produced in rabbit, bovine serum albumin (BSA), and 2,2,2-tribromoethanol were purchased from Sigma-Aldrich (St. Louis, MO). Hoechst 33342, Dulbecco’s modified Eagle medium (DMEM), Roswell Park Memorial Institute (RPMI)-1640 medium, 0.25% Trypsin-EDTA, Penicillin-Streptomycin (10,000 U/mL), 35 mm Glass Bottom Dishes and Fetal Bovine Serum were purchased from Thermo Fisher Scientific (Pittsburgh, PA, USA). Dulbecco’s phosphate-buffered saline (PBS) was purchased from Solarbio (Beijing, China). Purified anti-human CD54 Antibody (clone: R6.5), MC-VC-PAB-MMAE and MC-GGFG-DXd were obtained from MAbPlex (Yantai, China). Sulfo-Cy5.5 NHS ester was purchased from Xarxbio (Xian, China). Cell counting kit-8 (CCK-8, Cat#K1018) was purchased from ApexBio (Houston, TX, USA).

### Cell culture

Human CCA cell lines, HuCCT1 (Cat#CL-0725) and HCCC-9810 (Cat#CL-0095) were purchased from Procell (Wuhan, China), TFK-1 (Cat#BFN60808817) was purchased from Bluefbio (Shanghai, China), HuH28 (Cat#CTCC-003-073) was purchased from Meisen Chinese Tissue Culture Collections (Zhejiang, China), QBC939 and SK-ChA-1 were obtained from Guangzhou Medical University (Guangzhou, China). One human embryonic kidney HEK293T (Cat#CRL-3216) cells was purchased from American Type Culture Collection (Manassas, VA, USA). HuCCT1, HCCC-9810, HuH28 and QBC939 were cultured in RPMI-1640, TFK-1, SK-ChA-1, and HEK293T cells were cultured in DMEM, all were supplemented with 10% fetal bovine sera, 100 units/mL penicillin and 100 units/mL streptomycin. All cells were maintained in a humidified incubator at 37 °C with 5% CO_2_.

### Quantification of cell membrane protein expression

Surface protein expressions of human CCA cells and normal cells were evaluated by flow cytometry. 1 × 10^6^ cells were collected and rinsed twice in PBS, and then were blocked by 1% BSA in PBS for 30 min in an ice bath. After BSA blockage, cells were incubated with 1 µg PE-conjugated antibodies for 1 h at room temperature (RT), respectively. Next, cells were rinsed three times in PBS, resuspended in PBS. Then the geometric mean fluorescence intensity ratio (MFI) of 1 × 10^4^ cells in each sample were collected by CytoFLEX LX (Beckman Coulter) without special FACS gating strategies. Quantitative analysis of cellular antigen expression was determined with Quantum^TM^ Simply Cellular® microspheres (Bangs Laboratories), using the protocol provided by the manufacturer.

### Validation of ICAM1 expression in patient samples

Immunohistochemical (IHC) studies were conducted on paraffin-embedded human CCA tumors and para-cancerous tissues. This work was approved by and conducted under the Cancer Hospital of The University of Chinese Academy of Science Medical Research Ethics Board (IRB-2021-403). Waiver of informed consent was obtained because de-identified residual tumor tissues and corresponding adjacent tissues of 78 CCA patients were used from the pathology department of Cancer Hospital of The University of Chinese Academy of Science. Samples were stained by IHC with anti-ICAM1 antibody (1:200) under standard protocol and evaluated by a gastrointestinal pathologist with no knowledge of sample identity.

### Genomic analysis of ICAM1 in human CCA

The ICAM1 mRNA expression of human CCA tumors and normal bile duct tissues were analyzed using the UALCAN: The University of ALabama at Birmingham CANcer data analysis Portal (http://ualcan.path.uab.edu/). Gene expression data were generated from 36 CCA patient tumors and 9 human normal bile duct samples within the database of The Cancer Genome Atlas Program (TCGA). All genomic datasets used in our studies are publicly available in the UALCAN: The University of ALabama at Birmingham CANcer data analysis Portal (http://ualcan.path.uab.edu/).

### Visualizing membrane localization of ICAM1 in cells

Membrane localization of ICAM1 in cells determined by confocal immunofluorescent staining. 1 × 10^6^ cells were seeded in a 35 mm Glass Bottom Dishes with 2 mL cell culture medium incubated overnight at 37 °C. As incubation finished, cells were rinsed twice in PBS and then were blocked by 1% BSA in PBS for 30 min in an ice bath. After BSA blocking, cells were co-stained with 1 µg PE-conjugated anti-human ICAM1 antibodies for 1 h at RT and rinsed in PBS three times. Hoechst 33342 was used to stain cell nuclei for 10 min. Immunofluorescent stained samples were dealt with 4% paraformaldehyde for 15 min at RT. After rinsing, samples maintained in PBS and then examined in A1R HD25 confocal microscope (NIKON).

### Determining antibody targeting efficiency on CCA cells

Visualizing identification of ICAM1 antibody endocytosis on human CCA cells by confocal immunofluorescent staining. 1 × 10^6^ cells were seeded in a 35 mm Glass Bottom Dishes with 2 mL cell culture medium incubated overnight at 37 °C. After medium removed, cells were rinsed twice in PBS and then were blocked by 1% BSA for 30 min in an ice bath. After BSA blocking, cells were co-stained with 1 µg PE-conjugated anti-human ICAM1 antibodies for 1 h in an ice bath and then rinsed in PBS three times. After rinsing, samples were incubated at RT for 0 min, 30 min, 60 min, 120 min, and 240 min. Immunofluorescent stained samples were treated with 4% paraformaldehyde for 15 min, respectively, and then examined in A1R HD25 confocal microscope (NIKON).

Quantitative detection of the antibody internalization efficiency by flow cytometry. 1 × 10^6^ cells were collected and rinsed twice in PBS, and then were blocked by 1% BSA for 30 min in an ice bath. After blocking, cells were incubated with 1 µg purified anti-human ICAM1 antibodies for 1 h in an ice bath. Next, cells were rinsed twice in PBS, resuspended in PBS, and then incubated at RT for 0 min, 30 min, 60 min, 120 min, and 240 min, respectively. After incubating, 0.5 µg PE anti-mouse IgG1 antibodies were added to each sample and ice bathed for 30 min. Every sample was rinsed and MFI of each one was determined in CytoFLEX LX (Beckman Coulter). The ratio of internalization was calculated by the following formula: %internalized = {(MFI of anti-mouse IgG)_0 min_ − (MFI of anti-mouse IgG)_t min_}/(MFI of anti-mouse IgG)_0 min _× 100.

### Preparation and characterization of ICAM1 ADC

ICAM1-MMAE and ICAM1-DXd were prepared in GLP grade by MabPlex (Yantai, China) via covalently conjugate ICAM1 antibody (R6.5c, GeneScript) with ADC linker and payload combinations (MC-VC-PAB-MMAE or MC-GGFG-DXd) as described previously^36,37^. The DAR of ICAM1-MMAE and ICAM1-DXd was measured by using a hydrophobic interaction chromatography.

### In vitro cytotoxicity tests in cells

Human CCA cells (HuCCT1, HCCC-9810, HuH28, TFK-1, SK-ChA-1, and QBC939) and human normal epithelial cells (293T) were seeded in a 96-well plate at a density of 1 × 10^4^ cells per well and allowed to incubate overnight. Then cells were treated with two different ICAM1 ADCs (ICAM1-MMAE and ICAM1-DXd), one ICAM1 monoclonal antibody and one chemotherapy drug Gemcitabine with concentrations ranging from 0–10 µg/mL using a serial dilution factor of 10. After cells were cultured for another 72 h, CCK-8 reagent was added to each well and then incubated at 37 °C for 1 h. The plate was read at the absorbance wavelength of 450 nm using a Spark® multimode microplate reader (TECAN). Cell viability was determined by comparing the absorbance between drug-treated wells and drug-free wells.

### Biodistribution and treatment studies in vivo

Mouse studies presented in this study were performed according to the protocols approved by the Institutional Animal Care and Use Committee (IACUC) of Institute of Basic Medicine and Cancer, Chinese Academy of Sciences. Tumor-specificity and biodistribution studies in vivo were based on a CCA tumor (HuCCT1) xenografted nude mouse model. 2 × 10^6^ cells contained 50% Matrigel were implanted into the right abdomen flank of 4–6-week-old female nude mice subcutaneously. After tumors were formed 100–200 mm^3^ in volume, mice were randomly divided into three groups of five. Mice in groups were received intravenous injection of IgG-Cy5.5, ICAM1-Cy5.5, and ICAM1-MMAE-Cy5.5 at a dosage of 5 mg/kg weight. In vivo near-infrared (NIR) fluorescence imaging was performed on treated mice using an IVIS Lumina III (PerkinElmer) at 48 h post-injection. Then mice were anesthetized with 200 µL 2.5% 2,2,2-tribromoethanol per mouse by intraperitoneal injection. After anesthetized, mice were euthanized and removed their vital organs, including brain, heart, liver, lung, kidney, and spleen. NIR fluorescence intensities of separated organs and excised tumors were measured by IVIS Lumina III.

The in vivo therapeutic efficacy of ICAM1 ADCs was tested in two CCA models, including a CCA tumor (HuCCT1) xenografted model and a PDX model. For the CCA tumor (HuCCT1) xenografted model, HuCCT1 cells (2 × 10^6^ cells per mouse) contained 50% Matrigel were injected into the right abdomen flank of 4–6-week-old female nude mice subcutaneously. Tumors were allowed to develop for 4 weeks and then reached 50–100 mm^3^ in volume approximately. Then mice were randomly distributed in five groups of five, and dealt with different treatment of PBS, Gemcitabine, ICAM1 monoclonal antibody, ICAM1-MMAE or ICAM1-DXd in an equivalent dosage of 5 mg/kg every 3 days via tail vein injection, with a total of 4 injections. The tumor volume was measured by using a caliper every other day and was calculated according to the formula, *V* = *L* × *W*^2^ × π/6. The end point was reached 4 weeks after the start of treatment, mice were anesthetized with 2,2,2-tribromoethanol, then euthanized. Subcutaneous tumors were excised to measure the mass.

PDX model for CCA were obtained from Cancer Hospital of The University of Chinese Academy of Science. Tumor tissues were cut into 8 mm^3^ pieces and subcutaneously transplanted into the right dorsal flank of 4–6-week-old female nude mice. After xenograft tumors were stably formed to 50–100 mm^3^, mice were intravenously injected with different drugs (PBS, ICAM1 monoclonal antibody, Gemcitabine, ICAM1-MMAE and ICAM1-DXd) with the same dosage used in the CCA tumor (HuCCT1) xenografted model. The tumor volumes were monitored for 6 weeks. At the end point, mice were euthanized after anesthetized and subcutaneous tumors were excised to measure the mass.

### RNA-seq analysis

RNA extraction, cDNA library preparation, RNA sequencing, quality control, and transcriptome profiling of end point tumors in different treatment groups of PDX model (PBS, ICAM1 mAb, ICAM1 DXd, and ICAM1 MMAE, three repeated samples per group) were performed by LC-Bio Technologies (Hangzhou, China) Co., Ltd. Differential genes were analyzed using DESeq2 in R programming language. Advanced Heatmap Plots and Gene Set Enrichment Analysis (GSEA) of RNA-seq data were performed using the OmicStudio tools at https://www.omicstudio.cn.

### Statistical analysis

All of the experimental data were collected in triplicate unless otherwise noted and are presented as mean ± standard deviation. Statistical variance was calculated by using unpaired two-tailed Student’s t-test or two-way analysis of variance (ANOVA) with Bonferroni post hoc tests. *P* ≤ 0.05 was considered statistically significant. All statistical analysis was performed using Graphpad Prism 8 software.

### Reporting summary

Further information on research design is available in the Nature Research Reporting Summary linked to this article.

### Supplementary information

Supplementary Information

Reporting Summary

## Data Availability

The data generated in this study are available within the article and its supplementary information files. RNA sequencing data is freely available within the NCBI GEO database (GSE233818).
