# Supplementary material for: Development of potent antibody drug conjugates against ICAM1+ cancer cells in preclinical models of cholangiocarcinoma
Source: NPJ Precis Oncol. 2023 Sep 16;7:93. doi: 10.1038/s41698-023-00447-z (PMC10505223; doi:10.1038/s41698-023-00447-z)

Supplementary Figure 1. DAR characterization of two constructed ICAM1 ADCs including ICAM1-DXd (a) and ICAM1-MMAE (b) by hydrophobic interaction chromatography (HIC).

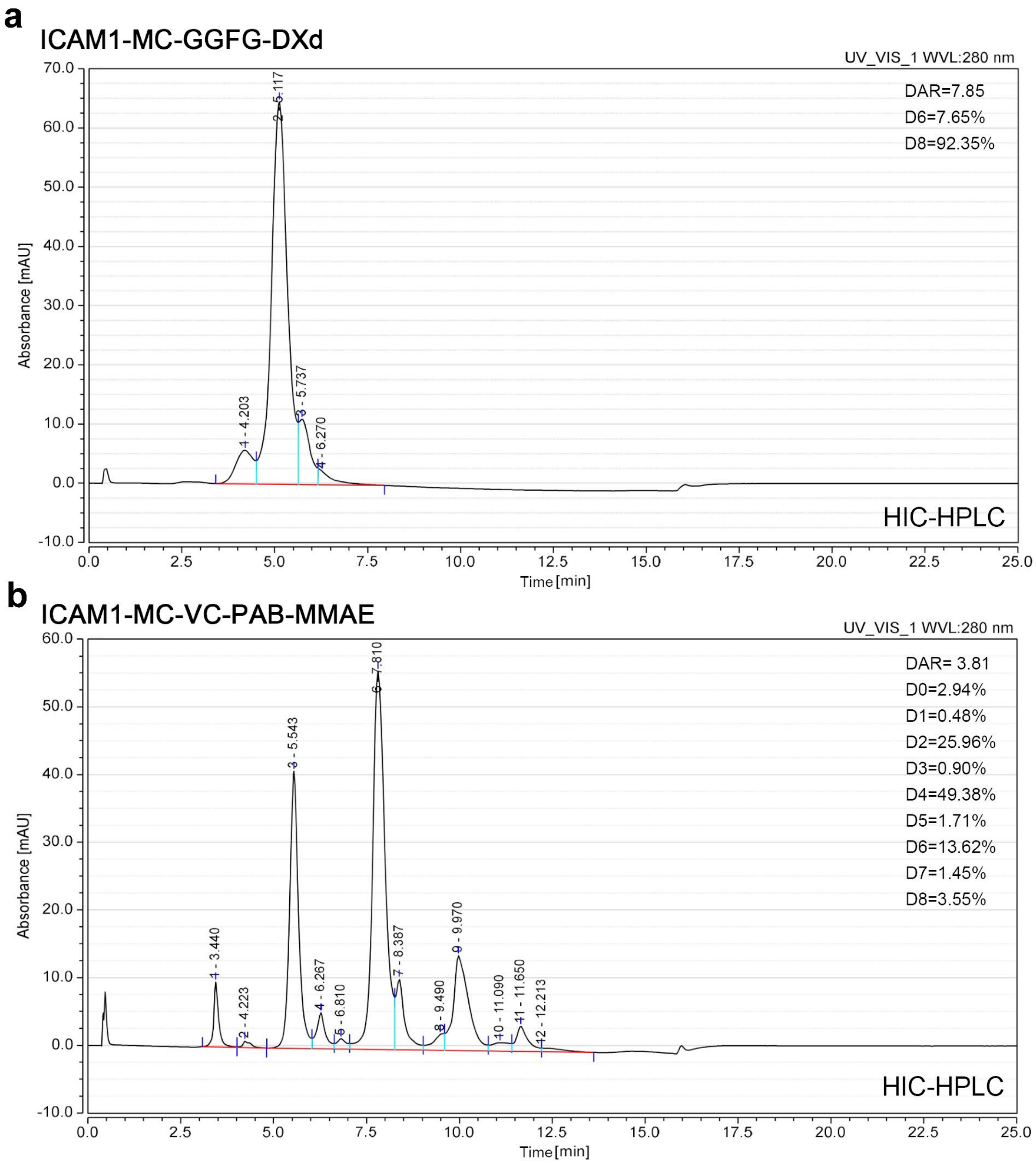

Supplementary Figure 2. Flow cytometry analysis of the binding ability of human ICAM1 monoclonal antibody (ICAM1 mAb), ICAM1-DXd and ICAM1-MMAE to two CCA cells. SK-ChA-1 (**a**) and TFK-1 (**b**). Cells were incubated with PBS, IgG, ICAM1 mAb, ICAM1-DXd or ICAM1-MMAE, respectively. After rinsing, primary antibody-stained cells were incubated with a secondary PE anti-human IgG, and determined by a flow cytometer.

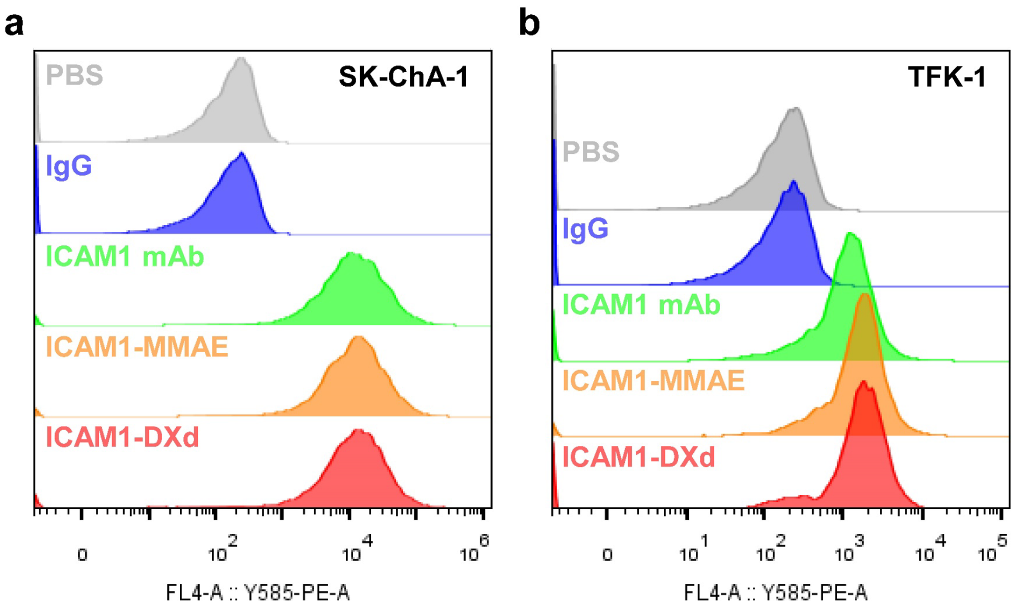

Supplementary Figure 3. The internalization of human ICAM1 monoclonal antibody (clone: R6.5) mediated by membrane protein ICAM1 on the surface of human CCA cells. **a** Representative IF images showing cellular internalization of ICAM1 mAb in human CCA cells (SK-ChA-1). Scale bar: 25 μm. **b** The internalization efficiency of ICAM1 mAb in four human CCA cell lines (QBC939, SK-ChA-1, TFK-1 and HCCC-9810) quantified by flow cytometry.

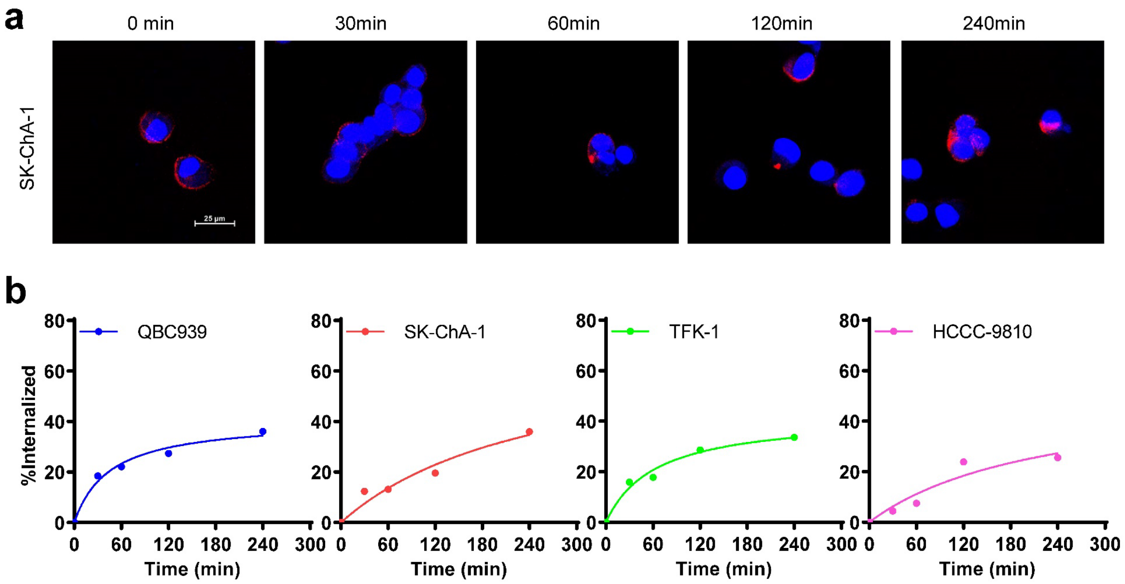

Supplementary Figure 4. Bystander killing effect of ICAM1 ADCs in co-culture conditions *in vitro*. TFK-1 (ICAM1$^+$) and RBE (ICAM1$^-$) cells were co-cultured at a ratio of 1:1 and treated with 10 μg/mL different ADCs for 6 days. After collecting adherent cells, cell number and ratio of TFK-1/RBE cells were determined by flow cytometry (**a**) and cell counting (**b**) analyses respectively. Each bar represents the mean and SD (N = 3).

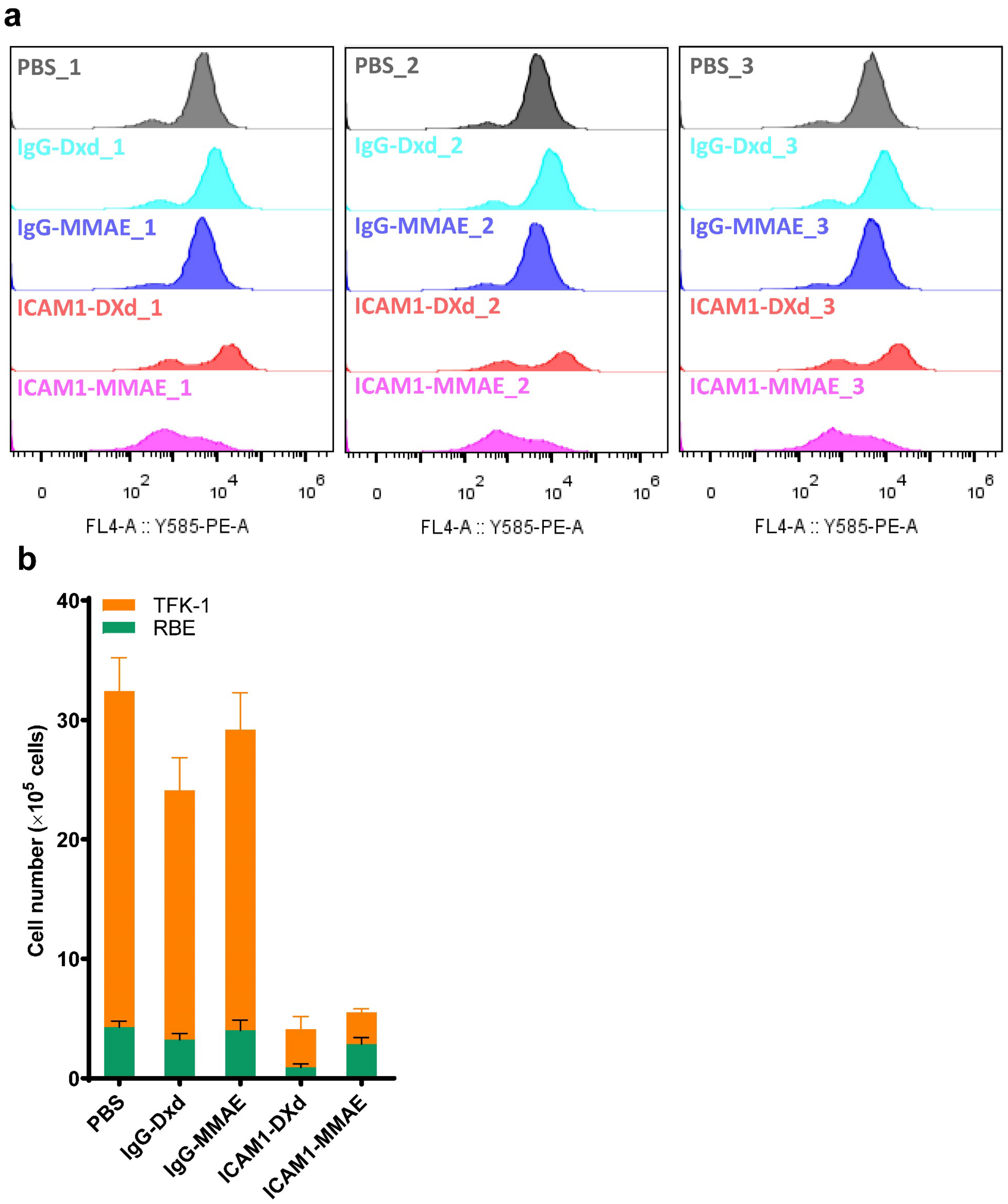

Supplementary Figure 5. Liver and renal toxicity analysis of ICAM1 ADCs in a CCA tumor (HuCCT1) xenografted model. a Blood chemistry. Each bar represents the mean and SD (N = 5). **b** H&E staining. Scale bar: 20 μm.

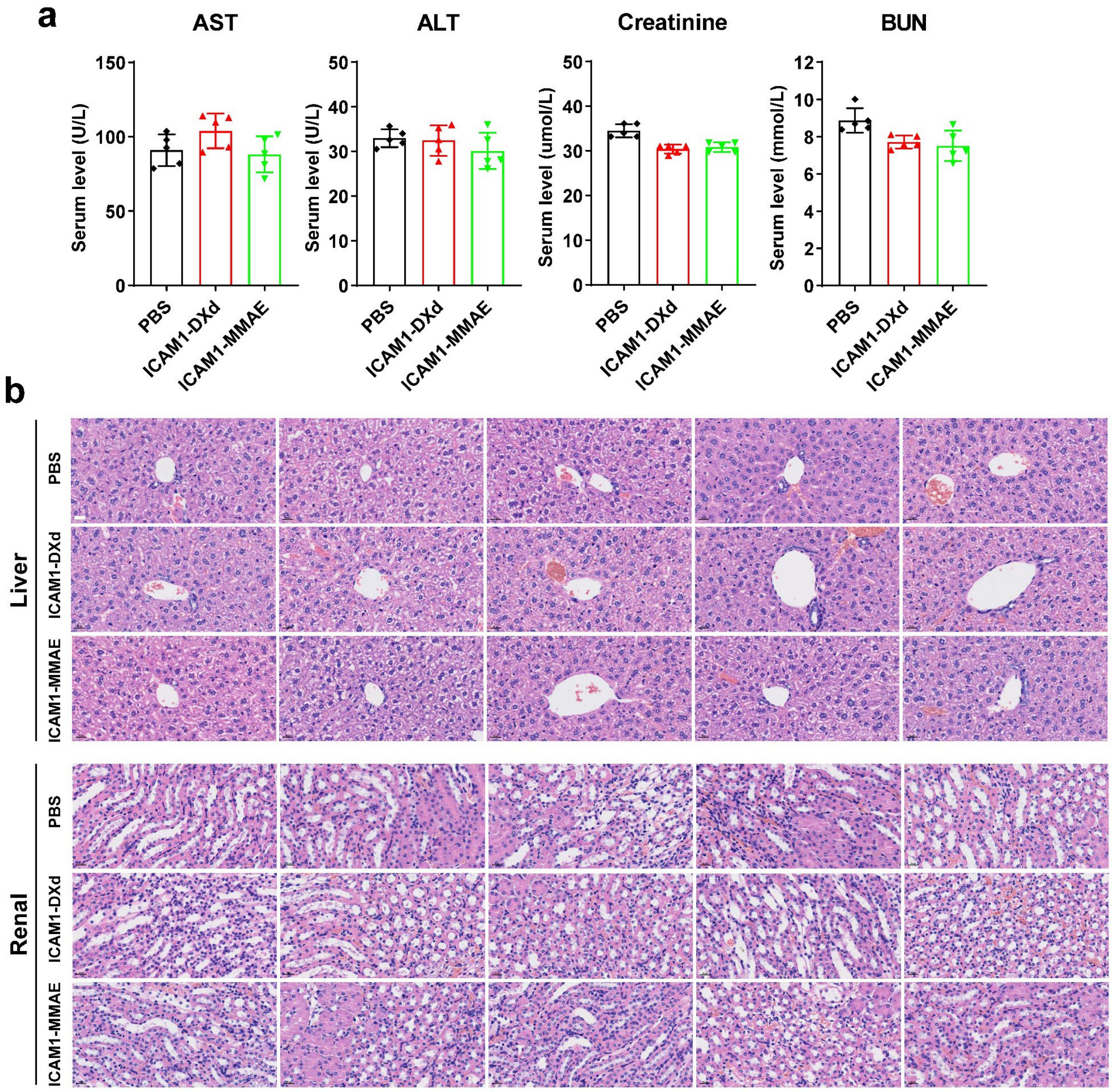

Supplementary Figure 6. Tumor-specific efficacy of ICAM1 ADCs in comparison with isotype ADCs on CCA tumors *in vivo*. a Schematic design of *in vivo* efficacy for ADCs in a CCA tumor (HuCCT1) xenografted model. **b** Tumor progression in mice received PBS, ICAM1-MMAE or IgG-MMAE, respectively, monitored by tumor volume measurement (N=5 per group). Two-way ANOVA, *P<0.05, **P<0.01. Error bars, SEM. **c** Image of excised HuCCT1 tumors from mice treated with PBS, ICAM1-MMAE or IgG-MMAE. Tumor mass (at day 18) (**d**) and mouse bodyweight (**e**) of mice received PBS, ICAM1-MMAE or IgG-MMAE, respectively. Error bars, SD. **f** Tumor progression in mice received PBS, ICAM1-DXd or IgG-DXd, respectively, monitored by tumor volume measurement (N=5 per group). Two-way ANOVA, *P<0.05, **P<0.01. Error bars, SEM. **g** Image of excised HuCCT1 tumors from mice treated with PBS, ICAM1-DXd or IgG-DXd. Tumor mass (at day 18) (**h**) and bodyweight (**i**) of mice received PBS, ICAM1-DXd and IgG-DXd. Error bars, SD.

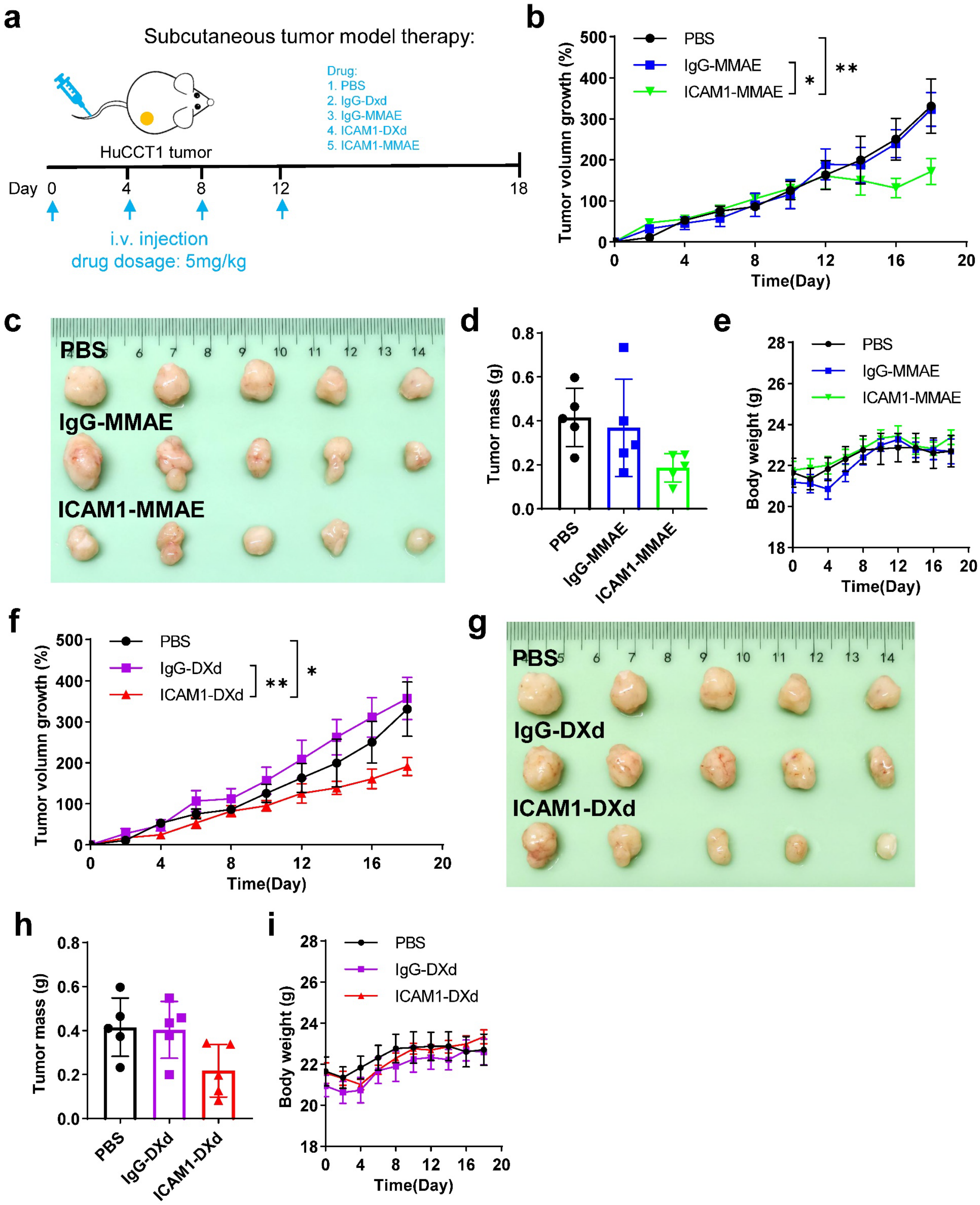
a
Subcutaneous tumor model therapy:
HuCCT1 tumor
Drug:
1. PBS
2. IgG-Dxd
3. IgG-MMAE
4. ICAM1-DXd
5. ICAM1-MMAE
Day 0 4 8 12 18
i.v. injection
drug dosage: 5mg/kg
b
Tumor volumn growth (%)
Time(Day)
PBS
IgG-MMAE
ICAM1-MMAE
*
**
c
PBS
IgG-MMAE
ICAM1-MMAE
d
Tumor mass (g)
PBS
IgG-MMAE
ICAM1-MMAE
e
Body weight (g)
Time(Day)
PBS
IgG-MMAE
ICAM1-MMAE
f
Tumor volumn growth (%)
Time(Day)
PBS
IgG-DXd
ICAM1-DXd
**
*
g
PBS
IgG-DXd
ICAM1-DXd
h
Tumor mass (g)
PBS
IgG-DXd
ICAM1-DXd
i
Body weight (g)
Time(Day)
PBS
IgG-DXd
ICAM1-DXd

Supplementary Figure 7. Flow cytometry analysis of ICAM1 expression level in the PDX model of CCA.

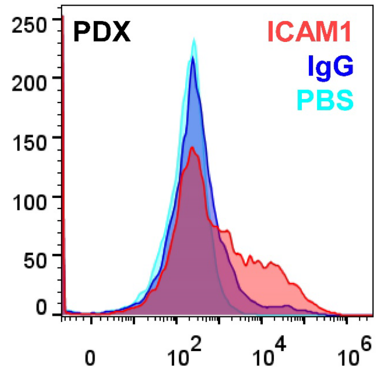

Supplement: Supplementary file 1 — Supplementary Information [file 41698_2023_447_MOESM1_ESM.pdf]
