# Supplementary material for: Development of potent antibody drug conjugates against ICAM1+ cancer cells in preclinical models of cholangiocarcinoma
Source: NPJ Precis Oncol. 2023 Sep 16;7:93. doi: 10.1038/s41698-023-00447-z (PMC10505223; doi:10.1038/s41698-023-00447-z)
Supplement: Supplementary file 2 — Reporting Summary [file 41698_2023_447_MOESM2_ESM.pdf]

nature portfolio

Corresponding author(s): Peng Guo & Jieer Ying

Last updated by author(s): Sep 1, 2023

Reporting Summary

Nature Portfolio wishes to improve the reproducibility of the work that we publish. This form provides structure for consistency and transparency in reporting. For further information on Nature Portfolio policies, see our Editorial Policies and the Editorial Policy Checklist.

Statistics

For all statistical analyses, confirm that the following items are present in the figure legend, table legend, main text, or Methods section.

n/a	Confirmed	
☐	☒	The exact sample size (*n*) for each experimental group/condition, given as a discrete number and unit of measurement
☐	☒	A statement on whether measurements were taken from distinct samples or whether the same sample was measured repeatedly
☐	☒	The statistical test(s) used AND whether they are one- or two-sided<br>*Only common tests should be described solely by name; describe more complex techniques in the Methods section.*
☒	☐	A description of all covariates tested
☒	☐	A description of any assumptions or corrections, such as tests of normality and adjustment for multiple comparisons
☐	☒	A full description of the statistical parameters including central tendency (e.g. means) or other basic estimates (e.g. regression coefficient) AND variation (e.g. standard deviation) or associated estimates of uncertainty (e.g. confidence intervals)
☐	☒	For null hypothesis testing, the test statistic (e.g. F, t, r) with confidence intervals, effect sizes, degrees of freedom and P value noted<br>*Give P values as exact values whenever suitable.*
☒	☐	For Bayesian analysis, information on the choice of priors and Markov chain Monte Carlo settings
☒	☐	For hierarchical and complex designs, identification of the appropriate level for tests and full reporting of outcomes
☒	☐	Estimates of effect sizes (e.g. Cohen's *d*, Pearson's *r*), indicating how they were calculated

Our web collection on statistics for biologists contains articles on many of the points above.

Software and code

Policy information about availability of computer code

Data collection	Collection of FACS data was performed using the software of CytExpert v2.4.0.28. Collection of CellSearch data was performed using the CytoFLEX LX (Beckman Coulter).
Data analysis	CytExpert software (Beckman Coulte) , FlowJo software, Graphpad Prism 8 software

For manuscripts utilizing custom algorithms or software that are central to the research but not yet described in published literature, software must be made available to editors and reviewers. We strongly encourage code deposition in a community repository (e.g. GitHub). See the Nature Portfolio guidelines for submitting code & software for further information.

Data

Policy information about availability of data

All manuscripts must include a data availability statement. This statement should provide the following information, where applicable:

- Accession codes, unique identifiers, or web links for publicly available datasets
- A description of any restrictions on data availability
- For clinical datasets or third party data, please ensure that the statement adheres to our policy

The data generated in this study are available within the article and its supplementary information files. RNA sequencing data is freely available within the NCBI GEO database (GSE233818).

Research involving human participants, their data, or biological material

Policy information about studies with human participants or human data. See also policy information about sex, gender (identity/presentation), and sexual orientation and race, ethnicity and racism.

Reporting on sex and gender	N/A
Reporting on race, ethnicity, or other socially relevant groupings	N/A
Population characteristics	N/A
Recruitment	N/A
Ethics oversight	N/A

Note that full information on the approval of the study protocol must also be provided in the manuscript.

Field-specific reporting

Please select the one below that is the best fit for your research. If you are not sure, read the appropriate sections before making your selection.

☒ Life sciences ☐ Behavioural & social sciences ☐ Ecological, evolutionary & environmental sciences

For a reference copy of the document with all sections, see nature.com/documents/nr-reporting-summary-flat.pdf

Life sciences study design

All studies must disclose on these points even when the disclosure is negative.

Sample size	All results are representative of experimental findings from experimental replicates of at least an n=3. All results were replicated using at least two model systems as indicated. Statistical comparisons analyzed in GraphPad Prism 8 using one-way or two-way analysis of variance (ANOVA) with Bonferroni post hoc tests
Data exclusions	No data were excluded.
Replication	All data was reproduced across at least two experiments and cell line model systems.
Randomization	Tumor bearing mice were randomly assigned to experiment groups and control groups.
Blinding	During experimental procedures investigators were not blinded.

Reporting for specific materials, systems and methods

We require information from authors about some types of materials, experimental systems and methods used in many studies. Here, indicate whether each material, system or method listed is relevant to your study. If you are not sure if a list item applies to your research, read the appropriate section before selecting a response.

Materials & experimental systems

n/a	Involved in the study	
☐	☒	Antibodies
☐	☒	Eukaryotic cell lines
☒	☐	Palaeontology and archaeology
☐	☒	Animals and other organisms
☒	☐	Clinical data
☒	☐	Dual use research of concern
☒	☐	Plants

Methods

n/a	Involved in the study	
☒	☐	ChIP-seq
☐	☒	Flow cytometry
☒	☐	MRI-based neuroimaging

Antibodies

Antibodies used	Phycoerythrin (PE)-conjugated mouse anti-human antibodies against 72 cancer target candidates(CD90, Cat#328110; CD106, Cat#305806; CD62P, Cat#304906; CD50, Cat#330005; CD87, Cat#336906; CD202b, Cat#334206; CD195, Cat#321606; CD31,

	Cat#303106; CD254, Cat#347504; CD24, Cat#323206; CD371, Cat#353604; CD140b, Cat#323606; CD54, Cat#353106; CD126, Cat#352804; CD104, Cat#327808; CD62E, Cat#336008; SSEA-5, Cat#355204; SSEA-3, Cat#330312; HER-3, Cat#324706; CD105, Cat#323206; PSMA, Cat#342504; CD266, Cat#314004; CD51/61, Cat#304406; CD257, Cat#366506; CD152, Cat#349906; CD309, Cat#393004; CD203c, Cat#324606; CD227, Cat#355604; CD144, Cat#348506; CD66a/b/c, Cat#342304; TM4SF20, Cat#367204; CD192, Cat#357206; CD140a, Cat#323506; CD152, Cat#349906; CD274, Cat#329706; CD317, Cat#127104; CD197, Cat#353204; CD117, Cat#375206; CD181, Cat#320608; CD141, Cat#344104; Notch 3, Cat#345406; CD70, Cat#355104; CD271, Cat#345106; FOLR1, Cat#908304; CD171, Cat#371604; EphA2, Cat#356804; SSEA-4, Cat#330406; CD326, Cat#324206; CD325, Cat#350805; CD44, Cat#397504; VEGFR-3, Cat#356204; CD46, Cat#352402; CD184, Cat#306506; ROR1, Cat#357804; CD340, Cat#324406; CD49c, Cat#343803; CD221, Cat#351806; EGFR, Cat#352904; CD324, Cat#324406; CD146, Cat#361006; CD304, Cat#354504; CD49a, Cat#328304; CD107a, Cat#328608; CD49b, Cat#359308; CD166, Cat#343904; CD56, Cat#362508; CD47, Cat#323108; CD49e, Cat#328010; CD276, Cat#351004; CD29, Cat#303004; CD9, Cat#312106; CD71, Cat#334106), PE mouse IgG1 (Cat#400114), purified anti-human CD54 Antibody (Cat#322702), PE anti-mouse IgG1 Antibody (Cat#406608) and PE anti-human IgG Fc (Cat#410708) were purchased from BioLegend (San Diego, CA, USA). Purified anti-human CD54 Antibody (clone: R6.5)were obtained from MAbPlex (Yantai, China).
Validation	All antibodies used were validated by manufacturer, used as per recommended protocols, and tested for each application using appropriate controls.

Eukaryotic cell lines

Policy information about cell lines and Sex and Gender in Research

Cell line source(s)	Human CCA cell lines, HuCCT1 and HCCC-9810 were purchased from Procell (Wuhan, China), TFK-1 was purchased from Bluefbio (Shanghai, China), HuH28 was purchased from Meisen Chinese Tissue Culture Collections (Zhejiang, China), QBC939 and SK-ChA-1 were obtained from Guangzhou Medical University (Guangzhou, China). One human embryonic kidney HEK293T cells was purchased from American Type Culture Collection (Manassas, VA, USA).
Authentication	Cell lines were authenticated using STR analysis.
Mycoplasma contamination	We did not test mycoplasma contamination in our cell culture.
Commonly misidentified lines (See ICLAC register)	No commonly misidentified cell lines were used in the study.

Animals and other research organisms

Policy information about studies involving animals; ARRIVE guidelines recommended for reporting animal research, and Sex and Gender in Research

Laboratory animals	4-6 weeks old female nude mice
Wild animals	No wild animals were used in the study.
Reporting on sex	Only female mice were used
Field-collected samples	At experimental endpoint, tumors were excised to measure the mass, and saved in 4% Paraformaldehyde.
Ethics oversight	Mouse studies presented in this study were performed according to the protocols approved by the Institutional Animal Care and Use Committee (IACUC) of Institute of Basic Medicine and Cancer, Chinese Academy of Sciences.

Note that full information on the approval of the study protocol must also be provided in the manuscript.

Flow Cytometry

Plots

Confirm that:

- [x] The axis labels state the marker and fluorochrome used (e.g. CD4-FITC).
- [x] The axis scales are clearly visible. Include numbers along axes only for bottom left plot of group (a 'group' is an analysis of identical markers).
- [ ] All plots are contour plots with outliers or pseudocolor plots.
- [x] A numerical value for number of cells or percentage (with statistics) is provided.

Methodology

Sample preparation	Cells were collected and rinsed twice in PBS, and then were blocked by 1% BSA in PBS for 30 min in an ice bath. After BSA blockage, cells were incubated with PE-conjugated antibodies for 1 h at room temperature (RT), respectively. Next, cells were rinsed three times in PBS, resuspended in PBS, and the geometric mean fluorescence intensity ratio (MFI) of each sample was determined in CytoFLEX LX (Beckman Coulter).

Instrument	CytoFLEX LX (Beckman Coulter)
Software	CytExpert v2.4.0.28, FlowJo v10
Cell population abundance	1x10^6 cells in PBS suspension. cell number and viability was determined by cell counter.
Gating strategy	Intact cells were gated according to the FSC-A and SSC-A. The geometric mean fluorescence intensity ratio (MFI) of 1x10^4 cells in each sample were collected by CytoFLEX LX (Beckman Coulter) without special FACS gating strategies.

☐ Tick this box to confirm that a figure exemplifying the gating strategy is provided in the Supplementary Information.
